# A Predictive and Adaptive Virtual Exposure Framework for Spider Fear: A Multimodal VR-Based Behavioral Intervention

**DOI:** 10.3390/healthcare13202617

**Published:** 2025-10-17

**Authors:** Heba G. Mohamed, Muhammad Nasir Khan, Muhammad Tahir, Najma Ismat, Asma Zaffar, Fawad Naseer, Shaukat Ali

**Affiliations:** 1 Department of Electrical Engineering, College of Engineering, Princess Nourah Bint Abdulrahman University, P.O. Box 84428, Riyadh 11671, Saudi Arabia; 2 Department of Electrical Engineering, Government College University Lahore, Lahore 54000, Pakistan; 3 Computer Software Engineering Department, Sir Syed University of Engineering and Technology, Karachi 75300, Pakistan; mtahira@ssuet.edu.pk; 4 Department of Computer Engineering, Sir Syed University of Engineering and Technology, Karachi 75300, Pakistan; nismat@ssuet.edu.pk; 5 Department of Mathematics and Sciences, Sir Syed University of Engineering and Technology, Karachi 75300, Pakistan; aszafar@ssuet.edu.pk; 6 Department of Computer Science and Software Engineering, Beaconhouse International College, Faisalabad 38000, Pakistan; fawad.naseer@bic.edu.pk; 7 Office of the Registrar, Government College University Lahore, Lahore 54000, Pakistan; drshaukat373@gmail.com

**Keywords:** virtual reality exposure therapy (VRET), arachnophobia, adaptive behavioral intervention, fear of spiders questionnaire (FSQ), physiological signal monitoring, AR-GARCH modeling, affective computing, real-time scenario adaptation

## Abstract

Background: Exposure therapy is an established intervention for treating specific phobias. This study evaluates a Virtual Exposure Therapist (VET), a virtual reality (VR)-based system enhanced with artificial intelligence (AI), designed to reduce spider fear symptoms. Methods: The VET system delivers three progressive exposure scenarios involving interactive 3D spider models and features an adaptive relaxation mode triggered when physiological stress exceeds preset thresholds. AI integration is rule-based, enabling real-time adjustments based on session duration, head movement (degrees/s), and average heart rate (bpm). Fifty-five participants (aged 18–35) with self-reported moderate to high fear of spiders completed seven sessions using the VET system. Participants were not clinically diagnosed, which limits the generalizability of findings to clinical populations. Ethical approval was obtained, and informed consent was secured. Behavioral responses were analyzed using AR(p)–GARCH (1,1) models to account for intra-session volatility in anxiety-related indicators. The presence of ARCH effects was confirmed through the Lagrange Multiplier test, validating the model choice. Results: Results demonstrated a 21.4% reduction in completion time and a 16.7% decrease in average heart rate across sessions. Head movement variability declined, indicating increased user composure. These changes suggest a trend toward reduced phobic response over repeated exposures. Conclusions: While findings support the potential of AI-assisted VR exposure therapy, they remain preliminary due to the non-clinical sample and absence of a control group. Findings indicate expected symptom improvement across sessions; additionally, within-session volatility metrics (persistence/half-life) provided incremental predictive information about later change beyond session means, with results replicated using simple volatility proxies. These process measures are offered as complements to standard analyses, not replacements.

## 1. Introduction

Fear occurs when a person feels threatened by certain phenomena or situations, but if a person is overwhelmed by that situation, then it is a phobia. Phobia is a Latin and Greek word for fear, and it is classified as a group of anxiety disorders. According to the *Diagnostic and Statistical Manual of Mental Disorders: DSM-5* [1], a phobia can be categorized into specific phobia, social phobia, and agoraphobia. Some of the symptom of phobia experiencing it are breathing difficulty, faintness, and in some cases severe panic attacks. Studies have shown that phobias are more prevalent in women than in men. Statistics have shown that around 10% of the world population have experienced phobia in some manner in their life [2], and it is twice as high in women as in men, according to the National Institute of Mental Health (NIMH) [3]. In this work, our focus is on the sub-category of specific phobia known as arachnophobia or the fear of spiders.

Human beings possess strong emotions for animals, whether it is love, fear, happiness, or inquisitiveness about their evolution, habitat, etc. [4,5]. Spiders inhabited the earth approximately 450 million years ago, and they are among the ones that trigger fear in humans and are one of the most common types of phobias in humans [6]. Arachnophobia (spider phobia) comes from the Greek words “arachne” (spider) and “phobos” (phobia). It is the disliking of spiders and other arachnoid and Phalangium opilio-like scorpions [7]. Most of the phobic patients do not take any treatment, and every one out of three women and one out of every four men suffer from this phobia.

Beyond documenting symptom change, the present study advances (i) a process-analytic view of exposure by quantifying within-session volatility (persistence, half-life) in multi-sensor signals and (ii) a transparent physiology-aware adaptation that can be implemented on commodity/standalone VR. These additions aim to (a) surface how exposure unfolds moment-to-moment, (b) identify early markers of response, and (c) provide replicable rules that therapists and developers can adopt without opaque black-box controllers.

**Figure 1:**
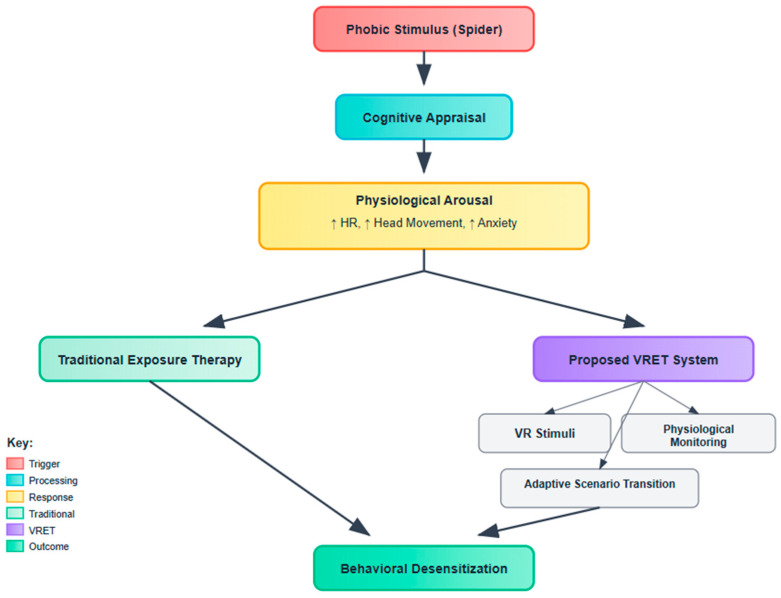
Conceptual framework showing how the VRET system mediates behavioral desensitization in spider-fearful individuals through staged exposure and AI-based adaptive feedback.

To contextualize the therapeutic pathway underlying spider fear treatment, Figure 1 outlines the cognitive behavioral framework employed in both traditional and virtual exposure paradigms. The model illustrates the process initiated by a phobic stimulus (e.g., spider), which elicits cognitive appraisal and subsequent physiological arousal—manifested as elevated heart rate, increased head movement, and heightened anxiety. While conventional exposure therapy addresses these symptoms through controlled real-world interactions, the proposed VRET system enhances this approach via immersive VR stimuli, real-time physiological monitoring, and adaptive scenario transitions. This integration is designed to facilitate behavioral desensitization through a safer, scalable, and adaptive modality.

To treat patients suffering from a phobia, one technique is exposure therapy. This technique falls in the category of psychological treatment of behavioral therapy. It is a scientifically proven technique where treatment is administered in a controlled manner by exposing the patient to the fearful environment and helping them avoid all possible ways to increase or intensify the fear [8,9,10,11]. Phobic disorder exposure therapy is the combination of classical techniques of exposure in vivo and in sensu [12,13] and is regarded as an effective way of treatment. The treatment methodology is to expose the patient to the feared situation that can increase the anxiety level, but in a virtual reality (VR) scenario, the patients can learn how to overcome their fears by exposing themselves to virtual stimuli that trigger the fear [14,15].

### 1.1. What Is New and Why It Matters

Prior VRET studies for spider fear have primarily reported pre–post outcomes and session mean, with limited visibility into within-session dynamics and heterogeneous—often implicit—rules for pacing and difficulty. The present work contributes three integrated advances: (i) a process-analytical layer that quantifies moment-to-moment settling (volatility persistence/half-life) across heart rate, head movement variability, and time-on-task using AR(p)–GARCH (and model-agnostic rolling/EWMA proxies); (ii) a transparent, adaptive relaxation environment—a rule-based controller that issues brief micro-relaxation prompts and step-up/hold decisions from pre-specified physiological/behavioral thresholds; and (iii) an AI-assisted scenario adaptation loop that transforms those thresholds into auditable difficulty updates (stimulus proximity, motion, and interaction pacing) on standalone hardware. Together, these elements standardize measurement and control logic so that exposure is not only effective but legible, reproducible, and portable across settings.

Like the traditional exposure therapy technique, researchers are working on a new therapeutic paradigm that involves exposure to the phobia’s virtual stimulus in a controlled environment. In in vivo therapy of spider fear, phobic patients are exposed to the real stimulus in a series of steps [16]. This treatment is successful but distressing for some patients. A better approach to the simulator, such as training, is involved in many areas of professional training/learning skills and in the treatment of anxiety disorders and preventive care [17,18]. For any imaginary stimulus, whether VR or augmented reality (AR), the patient interacts with a three-dimensional (3D) environment where the phobic stimulus is introduced in steps [19]. Some of the VR-based therapeutic treatments are available on the market, but researchers are working on developing new solutions to deal with different types of phobias and studying the efficacy and effectiveness of VRET treatments in contrast to in vivo therapy. Some of the benefits of VRET are that psychologists have more control of the patient during the treatment session by adjusting the treatment during the session based on the patient’s anxiety levels, as well as reduced treatment time at a reasonable cost [20,21,22]. The need for new virtual treatment also increased with the emergence of the COVID-19 pandemic in 2019. During the lockdown period and after that, there was a sudden increase in patients suffering from anxiety disorders. So, there are some mobile self-help solutions and a combination of techniques like telemental health (TMH) [23], and VRET [24] is also proposed.

VRET exposure therapy involves the use of technology to treat patients suffering from different types of phobias. The participants of the VET treatment are challenged in a fear environment with a series of computer-generated virtual stimuli so that they can overcome their fear [25,26]. This work focuses on providing VRET exposure therapy treatment to patients suffering from spider fear. The proposed design provides a safe and realistic 3D environment where the therapist and the patient can interact with spiders.

### 1.2. Prior Work and Remaining Gaps

Over two decades of trials show that VRET can reduce specific-phobia symptoms, and—critically for dissemination—automated VRET has demonstrated non-inferiority to in vivo exposure in spider phobia (randomized trial) and strong effects in fully automated fear-of-heights interventions guided by a virtual coach. More recent effectiveness work extends delivery to consumer and smartphone platforms (e.g., oVRcome) and to therapist-guided mobile AR exposure for spider phobia, underscoring a trend toward scalable, low-touch implementations. However, despite these advances, field-level assessments highlight variable methodological quality and limited standardization of protocols, outcome timing, and process measurement, which complicate inference about mechanisms and personalization.

Parallel developments in AI-assisted and biofeedback-enhanced exposure suggest the promise of closed-loop titration using psychophysiological and behavioral signals (e.g., heart rate/HRV, skin conductance, head/eye movement). Early protocols and trials have begun to adjust exposure in real time based on monitored arousal, and biofeedback adjuncts to self-guided VRET show reductions in perceived arousal—yet implementations remain heterogeneous, markers and thresholds are not standardized, and few studies provide transparent algorithms or formal analyses of within-session dynamics that could explain why and for whom adaptation helps. Contemporary overviews of biomarkers and VR stressor “dose–response” further argue for quantitative characterization of variability during exposure, rather than reliance on session means alone.

### 1.3. Rationale and Contributions

Although VRET is now supported by multiple trials and reviews for anxiety and specific phobias—including spider phobia—the next wave of work is shifting from demonstrating if VRET works to explaining how and for whom it works under scalable delivery models. Contemporary commentaries and meta-analytic syntheses underline two persistent gaps: first, most studies rely on pre–post outcomes or coarse session summaries, with relatively little attention to within-session process dynamics that may index inhibitory learning (e.g., variability and fluctuation patterns across an exposure); second, physiology-aware adaptation inside VR remains at an early stage, with heterogeneous implementations and limited standardization. Addressing these gaps is crucial for advancing mechanism-informed, personalized VRET and for improving clinical adoption.

In response, the present study contributes a process-analytical, multi-sensor framework and a pragmatic adaptive protocol. First, we move beyond mean levels to analyze moment-to-moment volatility in engagement/arousal using an autoregressive–GARCH family model applied to three continuous signals—time-on-task, head movement (behavioral avoidance/approach proxy), and heart rate—thus operationalizing within-session dynamics that mechanism papers argue are theoretically relevant to exposure learning. Second, we operationalize an adaptive relaxation trigger that gates brief down-regulation opportunities when physiological or behavioral thresholds are exceeded, aligning with recent calls for data-driven titration rather than static hierarchies. Third, we evaluate this within a standalone, accessible setup akin to emerging smartphone/standalone VR deployments, a direction emphasized for real-world scale-up. Collectively, these elements position our study not as a replication of VRET efficacy per se, but as an advance in process-level measurement and adaptive control, intended to inform mechanism, personalization, and implementation.

### 1.4. Alternative Mechanisms: A Perceptual Control Theory (PCT) View of Exposure

Beyond cognitive behavioral accounts that emphasize habituation and expectancy violation, exposure can also be understood through Perceptual Control Theory (PCT), which models behavior as the control of perception via goal-directed, closed-loop regulation. From a PCT perspective, avoidance and safety behaviors function as control actions that keep threat-relevant perceptions within preferred bounds; exposure succeeds when individuals learn to maintain goal-consistent control (e.g., approaching/remaining near spiders) while perceiving feared cues, thereby reducing conflict between higher-level goals (e.g., “stay safe” vs. “perform valued action”). Virtual reality exposure is especially compatible with this view because it affords moment-to-moment user control of intensity, proximity, and duration—precisely the parameters people adjust when controlling perception. In our protocol, user-titrated pacing and rule-based micro-relaxation instantiate a closed-loop control context: participants adjust their behavior in response to arousal changes, and the system adaptively gates difficulty, allowing for reorganization of control systems rather than mere fear reduction [27,28,29,30].

This PCT framing yields testable implications distinct from a purely mean-level CBT account: (i) outcomes should track improvements in perceived control/agency and goal-consistent performance under threat, not only reductions in average fear; (ii) within-session settling (shorter volatility half-life) reflects stabilization of control loops; and (iii) reduced reliance on safety behaviors/avoidant head movement patterns indexes better control rather than simply lower arousal. We therefore treat our volatility metrics and adaptive titration not as alternatives to CBT mechanisms but as complements that operationalize control-theoretic change in a self-administered VRET context.

Although participants were not clinically diagnosed with spider fear, prior studies have shown that even non-clinical populations exhibiting moderate fear of spiders can benefit from VR-based exposure paradigms for both therapeutic and experimental insight [31]. This study aims to evaluate generalized fear reduction, rather than clinical treatment efficacy alone.

The rest of this paper is organized as follows: Section 2 covers the background and literature review. The methodology and statistical analysis are discussed in Section 3 and Section 4. Results of the experimentation are discussed in Section 5, and Section 6 concludes the paper.

## 2. Literature Review

Exposure therapy is a well-established technique for treating patients suffering from phobias and anxiety disorders. It is a form of psychotherapy conducted either in real stimuli, known as in vivo exposure therapy, or in imagined stimuli, known as VR exposure therapy. A meta-analysis [32] found that VRET was superior to being on a waitlist and that outcomes were durable over time. Research has been done to see the impact of VRET when used for spider fear.

In [33], the authors developed and evaluated a self-help smartphone application (Zerophobia), which is prescribed for treating patients with spider fear. It follows Cognitive Behavioral Therapy (CBT) protocols, and the duration of therapy is six weeks. The CBT contents comprise 2D animations and voiceovers. Users can take therapy sessions at their own pace within a six-week period, and it is expected that their fear of spiders will be reduced after completion of the therapy. There are a total of 112 participants, and they are divided into intervention and waitlist groups. All participants must fill out the pre-therapy and post-therapy questionnaires for the evaluation. 

In [34], an E-PCG technique was proposed that acted on the user’s responses. For its implementation, virtual spiders and virtual subjects are used. The spider model changes using the Procedural Content Generation via Reinforcement Learning (PCGRL) technique. The model changes as per the stress levels defined by the therapist. Around 100 spider models are generated using different attributes, and the stress levels of the virtual participants are calculated, which is a function of spider attributes. Three stress levels (low, moderate, and high) are calculated for the initial and termination states, and it is found that the ideal attributes for each stress level are minimum, average, and maximum, respectively.

The researchers in [35] developed and evaluated a VR exposure system that showed a level decrease but did not indicate whether the change was real. The work focused on the impact of VRET on the increase and decrease in spider fear in undiagnosed patients with spider fear. Results showed that the VRET interventions both induced and reduced discomfort in the VR group. After experiencing VRET, the phobic patient’s discomfort level decreases, but that does not indicate whether it is real.

Research was conducted in [36] to study the avoidance behavior of people suffering from spider fear. The participants of the study were all female; they were divided into two main groups: arachnophobes and non-arachnophobes. The non-arachnophobes were further divided into subgroups of fearful and non-fearful. The VRET design contains one fear stimulus of a spider and a neutral animal. The CR setup tracks the movement pattern of the head, limb, and body along with heart rate, pupil size, and gaze behavior. The participant filled out a questionnaire that had questions from different evaluation techniques like the Big Five Inventory (BFI), Trait Anxiety (TAI), Fear of Spider Questionnaire (FSQ), Short Scale for the Assessment of Locus of Control, assessment of disgust sensitivity (FEE), etc. The collected data was analyzed with ANOVA. The results showed that arachnophobes exhibit a strong dislike of spiders in VR and significant avoidance behavior. The fearful and non-fearful subgroups both found the presence of a spider slightly more unpleasant than that of a neutral animal.

In [37], a technician-controlled VRET study of spider fear reported significant decreases at 12-month follow-up. In total, 100 people were randomized to the study. The participants were assessed on the behavioral approach test (BAT) and self-rated fear of spiders, anxiety, depression, and quality-of-life scales. Assessments were conducted before and after the session and were evaluated using the linear mixed model. Post-treatment meetings were conducted several times after 1 week, 3 months, and 12 months. Results showed that there was a substantial decrease in avoidance in both VRET and OST groups. Upon the post-treatment assessment conducted after one week, the in vivo exposure therapy was superior to VRET, and this superiority was maintained in follow-up assessments. The VRET treatment is not worse than in vivo one-session treatment (OST), but non-inferiority was identified after 3 months but significantly decreased when 12-month follow-up assessments were performed.

In [38], the VRET application was designed and studied; participants’ anxiety levels and sense of presence were assessed. The application is designed with ultrasonic, tactile feedback while encountering the virtual spiders. A total of 35 adults participated in the study, both male and female, and their fear of spiders was assessed using FSQ. The anxiety levels were noted using skin conductance, a subjective units of distress scale (SUDS), and electroencephalogram (EEG) of the participants who showed a middle level of fear of spiders. The evaluation was also conducted from the perspective of finding the relationship between the participant’s anxiety level and their sense of the virtual environment.

## 3. Materials and Methods

The application designed for treating spider fear patients has three scenarios, which are named stages. To record the patient’s behavior during this VR-based treatment, an AI-embedded 3D object is designed, and a Leap Motion handheld controller that tracks hand gestures is used. The proposed solution is controlled by a specialized operator to avoid any unwanted situations during the treatment. The patient’s behavioral history is maintained, and at the end of each treatment session, a report is generated. To minimize the concerns and stress levels of the patients taking the treatment, the application is designed like a game to be completed by the patient by passing different levels, just like a regular game. There are stages of the therapeutic application, and each has multiple levels. Patients were asked to fill out the questionnaire after the completion of the session. While this study used a repeated measures design without a control group, future work should incorporate a waitlist or placebo control to assess treatment-specific effects.

While previous research [39] has applied AR(p)–GARCH modeling to physical phenomena such as solar cycles, the current study is the first to adapt this volatility-aware approach to a behavioral psychology context—specifically, modeling physiological desensitization trajectories during AI-enhanced virtual exposure therapy. By integrating affective computing with time series volatility modeling, this research bridges a novel interdisciplinary gap between psychophysiological intervention and dynamic systems forecasting.

### 3.1. Participants

The study targeted adults with high spider fear rather than a clinician-diagnosed specific phobia cohort. Eligibility required (i) age ≥18 years; (ii) self-reported fear of spiders; and (iii) a score ≥ [FSQ ≥ 55 or SPQ ≥ 20] on a validated spider-fear instrument, thresholds that have been used to define clinically meaningful severity in recent VRET studies and screening research. Formal diagnostic interviews were not administered; thus, the sample should be interpreted as high-fear/non-diagnosed, consistent with prior mechanistic and accessibility-focused VRET investigations of spider fear. Not all participants were diagnosed with spider fear, but some exhibited a fear of spiders, and some just wanted to challenge themselves for the diagnosis. All participants signed an informed consent form prior to their involvement in the study, in compliance with the ethical guidelines approved by the institutional review board with the approval number (BIC/IRB-25-00183).

Participants were recruited via [channels], completed web-based prescreening (FSQ/SPQ), and underwent on-site confirmation prior to Session 1. For therapeutic anchoring, we captured a brief functional-impact item (“fear interferes with daily activities: yes/no”) and recorded prior treatment (CBT/medication: yes/no). Prior to immersion, all participants completed a VR-safety checklist; the simulator-sickness subscale was monitored post-session. Participants who discontinued due to adverse effects were recorded as non-tolerant and analyzed under intention-to-treat conventions.

Prior to participation, individuals completed the Fear of Spiders Questionnaire (FSQ) to assess their baseline levels of spider fear. A minimum FSQ score of 40 was used as a threshold for inclusion, ensuring that all participants exhibited at least moderate fear levels, even if not clinically diagnosed. This approach allowed classification into low, moderate, and high fear groups for comparative analysis. A formal control group was not included in this study due to logistical constraints and the exploratory nature of the system’s initial deployment. The primary goal was to assess behavioral and physiological changes over repeated exposures in a single-group repeated measures design. This design is commonly used in early-stage VR-based therapy research to evaluate within-subject treatment effects over time.

This study targeted individuals who self-reported a significant fear of spiders, as measured by the Fear of Spiders Questionnaire (FSQ ≥ 40), but who were not formally diagnosed with specific phobia by a clinician. The aim was to evaluate the VET system’s effectiveness in a general population context, focusing on potential use in educational, workplace, or self-help therapeutic settings. This aligns with the system’s intended application as a scalable, pre-clinical intervention tool rather than a direct substitute for clinical therapy.

We implemented a brief micro-relaxation trigger during exposure, specified in three parts so others can reproduce it:

1. Signals & baselining: heart rate (HR; 5-s median, z-scored to each participant’s pre-exposure baseline), and head-movement variability (HMV; 5-s rolling SD of head-pose velocity, z-scored to the first 2 min).
2. Trigger rule (checked each second): issue a 30–45 s breathing prompt and temporarily hold difficulty if HR z ≥ +1.0 for ≥10 s or HMV z ≥ +1.0 for ≥5 s. If ≥3 triggers occur within 5 min, sustain current difficulty for the remainder of the block; if no triggers occur for 5 min, step up one level.
3. User-visible state: on-screen cue + haptic pulse for 1 s, then a progress ring counting down the micro-relaxation.

### 3.2. Hardware and Software Components

#### 3.2.1. Hardware

The hardware used for the proposed work includes a desktop PC with a GTX-1070Ei graphics card, a Head-Mounted Display (HMD) HTC Vive, a Leap Motion controller for hand tracking, a pulse rate Sensor (Sen-11574) to check heart rate in beats per minute, a 3D stereo hand, and Tripods.

#### 3.2.2. Software

The spider fear virtual therapy application is designed in Visual Studio Version 17.14.15 using C#. The virtual scenarios are designed with the game engine Unity 3D and modelled and textured with Blender 2.79 b. For the generation of reports, Adobe Photoshop Version 25.x and Microsoft Visual Studio are used.

**Figure 2:**
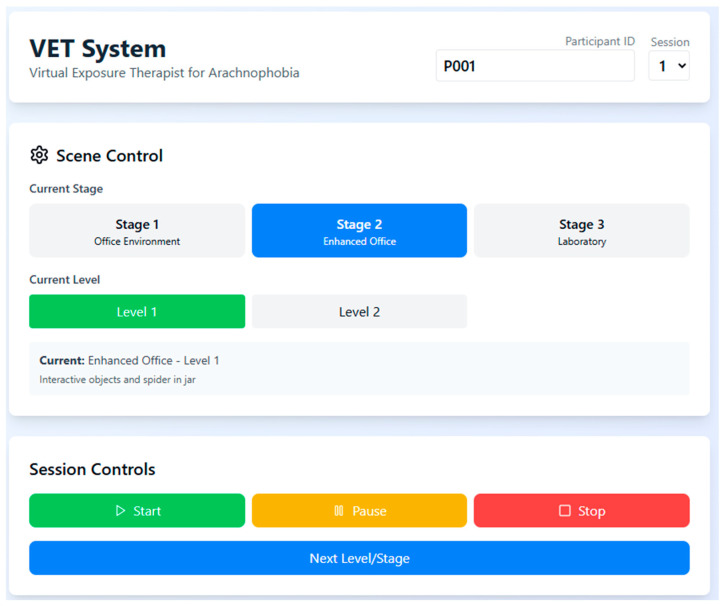
VET system interface showing participant progress tracking, physiological recording (heart rate, head movement), and session data reporting options including AR-GARCH analysis.

To monitor individual progression and performance during the VRET sessions, a real-time dashboard was used to capture key behavioral metrics such as heart rate, head movement, and time spent in the session. The system also tracked the number of relaxation triggers activated and provided stage-wise progress indicators, enabling both participants and researchers to assess treatment advancement and session completion status (Figure 2).

**Figure 3:**
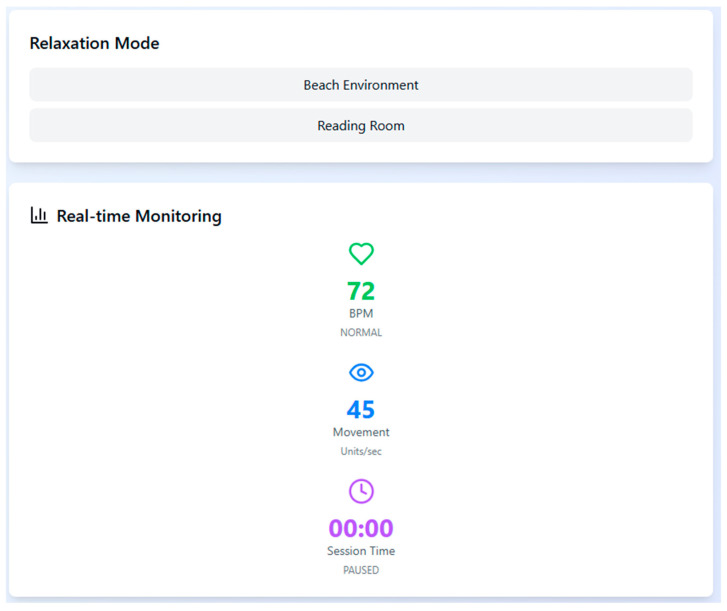
Real-time monitoring interface with relaxation mode options. The system tracks heart rate, head movement, and session duration to guide scenario transitions.

To support physiological stabilization during high arousal moments, the system includes two relaxation environments—Beach and Reading Room—which can be triggered automatically when the participant’s heart rate exceeds 120 bpm. These are accompanied by real-time monitoring of bio-signals to ensure session safety and to adapt stimuli responsively (Figure 3).

**Figure 4:**
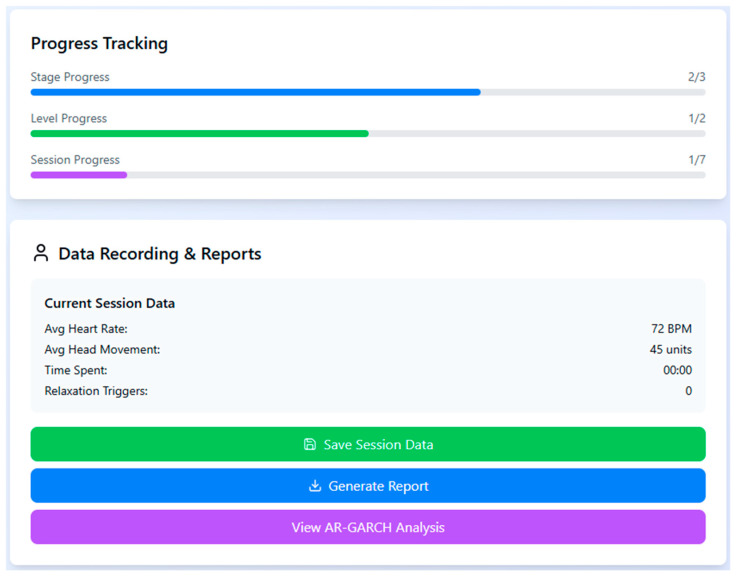
Scene control interface of the VET system, enabling stage/level selection and session management, including pause, start, and transition functionality.

A structured session control interface allows the operator to manually select stages and levels for each participant while tracking their progression and current scenario details. The interactive control panel enables pausing, stopping, or transitioning between levels to maintain therapeutic pacing and prevent overstimulation (Figure 4).

### 3.3. Treatment Scenarios

**Figure 5:**
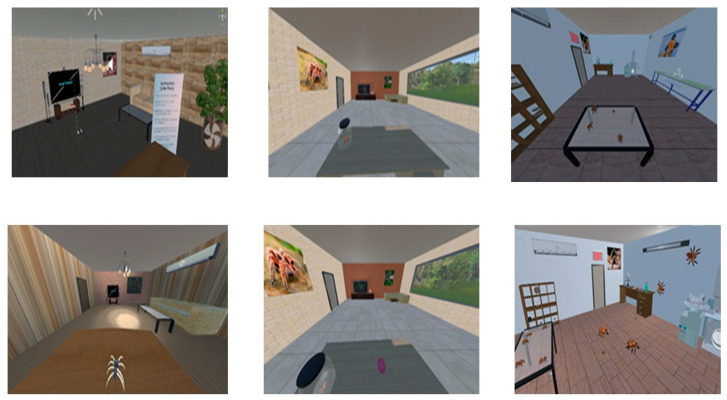
Virtual environments of arachnophobia.

The scenarios created for the patients suffering from spider fear are like a story-based game, shown in Figure 5. There are three stages, and each has multiple levels. To move from stage 1 to stage 3, the patient must pass all the levels of the stage. There are two additional scenarios to relax the patient while moving between stages, or if the patient’s heart rate is more than 120 bpm, then the VET application automatically changes from the spider fear scenario to the relaxed scenario. The heartbeat threshold is set according to an adult’s normal heartbeat, ranging between 60 and 120 BPM. The application has registration and reports generation modules to keep track of the patient’s therapy progress.

#### 3.3.1. Stage 1

This stage has two levels. The scene created for the first stage is an office with a window. The room is furnished with a chair, two tables, an air conditioner, and an LED TV with a picture of a spider on display. The room has a simple chandelier, a cartoonish spider, and a door to enter. In level 1, the patient wears the headset, and the application is started. The patient finds himself/herself in the office. This level is designed to familiarize patients by looking at pictures of tarantula spiders, and through hand gestures, patients can read instructions about Spider fear. After completing level 1, the next level is a modified version of the first, where an additional object, a robotic cartoon spider, is introduced to observe the behaviour of the patient and note down the fear level.

#### 3.3.2. Stage 2

The scene created for the second stage is also an office with a window with some additional objects, including a couch, a ball, and a spider in a glass jar. The room walls are lightly colored. This stage has two levels, namely level 3 and level 4. Level 3 is designed such that the patient can see outside the real environment through the office window. At level 4, some additional activities are introduced to the patient, such as interacting with the ball in the room.

#### 3.3.3. Stage 3

The scene created for the third stage is a laboratory. The laboratory walls are in a light gray colour and have a wooden floor. There is a table and chair, an air conditioner, a rack having glass jars full of spiders, and a framed picture on the wall. There is a bottle of chemicals and a file on the table. The laboratory has a washing sink as well. It has three levels, namely 5, 6, and 7.

At level 5, a window is designed such that the patient can see outside the real environment through the window. A video of the spider is playing on the TV, and to view the jar of spiders by pressing the F12 key, the patient can view or zoom in on it. After completing this level, the patient enters level 6, which has been designed with special sound effects so that spiders can sense human presence and they can hide. This level is designed keeping in mind that spiders also habitually stay away from humans, and they sense the sound when humans breathe, snore, and even their heartbeats.

#### 3.3.4. Relaxed Stage

There are two VR scenarios designed to make the patient comfortable when they move from one stage to another stage and at any time during the session, if the patient’s heart rate is more than 120 bmp (set according to an adult’s normal heartbeat, ranging between 60 to 120 bmp), then the application automatically changes the phobia environment to a relaxed environment.

The first relaxation scenario is a beach where the natural sound of wind and water, the sound of a ship passing, is playing in the background. The second scenario is an indoor setup, a reading room with chairs, a table, and a bookshelf. The relaxing music is playing in the background.

### 3.4. Measures

To measure the progress of the therapy, the time (in seconds) spent by the participants during the experiment in each claustrophobic VEs for seven attempts is measured. At the end of each level, a report is generated that records the head movement, heart rate in bpm, and the time spent in seconds. The participants were also asked to answer four questions at the end of each session when they completed the assigned task.

Although the participants answered a post-session questionnaire designed by the researchers, the study did not include a validated psychometric instrument such as the Fear of Spiders Questionnaire (FSQ), Subjective Units of Distress Scale (SUDS), or Beck Anxiety Inventory (BAI). This limits the ability to compare outcomes against established clinical benchmarks for phobia and anxiety. In future studies, incorporating standardized self-report tools will enable more rigorous assessment of symptom severity and treatment efficacy, particularly in clinical or diagnostic populations. After each session, participants completed a study-specific post-session questionnaire (PSQ) consisting of brief Likert-type items targeting perceived anxiety, task tolerability, perceived presence/engagement, and momentary avoidance tendencies (e.g., urge to look away/step back). The PSQ was designed to monitor process and tolerability and to inform pacing; it is not a standardized clinical outcome measure and was not used to infer treatment efficacy.

After each session, participants completed a brief study-specific PSQ designed for process monitoring and pacing, not for clinical outcome inference. The PSQ comprised 15 items covering five domains: state anxiety, perceptual avoidance/approach, perceived control/agency, presence/immersion, and tolerability/side-effects. One single-item indicator captured step-up readiness. Items used 7-point Likert scales unless noted. Table S-PSQ lists full stems and coding.

- State anxiety (momentary; 0–100 SUDS-style):
  A1. “Right now, how intense was your fear or anxiety at the end of this session?” (0 = none, 100 = extreme)
  A2. “At the peak of this session, how intense was your fear or anxiety?” (0–100)
- Perceptual avoidance/approach (1–7):
  AV1. “I felt an urge to look away from the spider.” (1 = strongly disagree, 7 = strongly agree)
  AV2. “I avoided looking directly at the spider.” (1–7)
  AV3. “I wanted to step back or increase the distance.” (1–7)
  (Higher = more avoidance; reverse-code if you prefer a ‘lower is better’ index.)
- Perceived control/agency (1–7):
  C1. “I felt in control of the pace of exposure.”
  C2. “I could pause or continue when I needed.”
  C3. “I could have handled more intensity if needed.”
  (Higher = greater perceived control.)
- Presence/immersion (1–7):
  P1. “I felt present in the scene.”
  P2. “The spider felt realistic to me.”
  P3. “My attention stayed on the task.”
  (Higher = greater presence/engagement.)
- Tolerability/side-effects (1–7 + binary):
  T1. “This session was physically comfortable.” (1–7)
  T2. “I experienced nausea/dizziness.” (1–7; higher = worse; reverse-code for the Tolerability Index)
  T3. “I would do another session like this.” (1–7)
  AE. “Any adverse effects?” (yes/no + free text)
- Step-up readiness (decision aid; 1–7):
  R1. “I feel ready to increase difficulty next time.” (1–7)

We computed domain scores as the mean of items within each domain (after reverse-coding AV2/AV3 and T2 where applicable):

- Anxiety Index: A1 (end) and A2 (peak) summarized descriptively (0–100); not combined with Likert items.
- Avoidance Index: mean (AV1–AV3), higher = more avoidance.
- Control Index: mean(C1–C3), higher = more perceived control/agency.
- Presence Index: mean(P1–P3), higher = greater presence/engagement.
- Tolerability Index: mean (T1, rev-T2, T3), higher = better tolerability.
- Readiness Indicator: R1 as a single decision-support item (≥5 suggests readiness, contingent on Tolerability ≥ 5 and no AE).

PSQ composites were used only as process indicators (session-to-session trends; exploratory associations with behavioral/physiological metrics). They were not treated as validated clinical endpoints. Clinical change should be inferred from validated instruments (e.g., FSQ/SPQ at baseline/post) and behavioral anchors (e.g., BAT) in future trials.

Within a single exposure, physiological and behavioral responses often arrive in bursts—periods of relative calm interspersed with spikes in arousal/avoidance—so the variance itself changes over time (i.e., conditional heteroskedasticity). Capturing this moment-to-moment volatility complements mean-level summaries and aligns with psychophysiology guidance to report dynamic characteristics of HR/HRV and related signals. We therefore model conditional variance to quantify how quickly arousal fluctuations persist or settle, operationalized as volatility persistence (α + β) and its half-life.

Although ARCH/GARCH originated in econometrics, analogous volatility-clustering phenomena are documented in biological signals. Prior work has used ARFIMA/ARMA–GARCH to characterize heart-rate variability and to detect volatility structure in epileptiform EEG, supporting the suitability of conditional-variance models for physiological/behavioral time series. Our use of AR(p)–GARCH follows these precedents and is aimed at describing within-session dynamics, not financial prediction.

### 3.5. Model-Utility and Added-Value Plan

1. Precondition: Fit AR(p) by BIC; apply ARCH-LM to AR residuals. Proceed to GARCH only if heteroskedasticity is detected (α_ARCH-LM_ ≤ 0.05).
2. Fit gain: Compare AR vs. AR-GARCH by AIC/BIC; report ΔAIC/ΔBIC and removal of residual volatility (ACF/Ljung–Box on squared residuals).
3. Incremental validity: In mixed-effects models predicting clinical change, enter session means first, then add volatility metrics (persistence α + β, half-life) or proxy measures (rolling SD/EWMA); report ΔAIC, ΔR^2^ (marginal/conditional), and LR-tests.
4. Transportability: Replicate key results with proxy volatility (no time series model), to show findings are model-agnostic.
5. Interpretation: Emphasize half-life (time to halve volatility after a spike) as an intuitive marker of settling; avoid parameter minutiae in main text.

## 4. Mathematical Model and Statistical Analysis

Traditional models like repeated measures ANOVA assume constant variance, which was not observed in this study’s time series data (time spent, head movement, heart rate). Instead, early sessions showed greater variability, stabilizing over time as participants habituated. To account for this volatility clustering and non-constant variance, the AR(p)–GARCH (1,1) model was employed. Its suitability for modeling dynamic physiological responses makes it appropriate for capturing emotional desensitization patterns in affective computing contexts.

We benchmark AR(p)–GARCH against standard approaches that target means rather than conditional variance:

1. Repeated measures ANOVA (RM-ANOVA) on session-level summaries (e.g., mean HR, mean head-movement, time-on-task), with Greenhouse–Geisser/Huynh–Feldt corrections when sphericity is violated.
2. Linear mixed-effects models (LMMs) with random intercepts (participant) and random slopes (session), and an AR(1) residual correlation when supported by diagnostics; where appropriate, we also fit a GAMM to allow smooth, non-linear trends across sessions.
3. Volatility proxies that require no time-series modeling (rolling SD and EWMA variance; 30–60 s windows).

For each signal and session, we then fit AR(p)–GARCH(1,1) only if ARCH-LM on AR residuals is significant. Model utility is evaluated by (i) AIC/BIC vs. AR-only alternatives; (ii) residual diagnostics (ACF of squared residuals; Ljung–Box); and (iii) incremental validity: adding volatility metrics (*α* + *β*, half-life, or proxy SD/EWMA) to LMMs predicting clinical or behavioral outcomes beyond session means (ΔR^2^/ΔAIC and likelihood ratio tests). This design treats RM-ANOVA/LMM as between-session estimators and AR(p)–GARCH as a within-session process measure; they address different questions and can be jointly interpreted.

### 4.1. Test Diagnostic

To verify the presence of the ARCH effect, Lagrange Multiplier (LM) tests and correlogram squared residuals were computed. Normality tests were applied to assess the feasibility of GARCH modeling. Model accuracy was evaluated using RMSE, MAPE, and MAE. Gaussian quasi-maximum likelihood estimation (GQMLE), commonly used in GARCH models for managing heavy-tailed returns [40,41], was employed due to its near-normal distribution and lower variance. Model selection was guided by AIC, BIC, and HIC values, while predictive adequacy was confirmed using RMSE, MAE, and MAPE. Definitions of these metrics are provided below.

Akaike information criterion (AIC) is the development of the maximum likelihood principle. The best-fitted model selection criterion consists of the smallest AIC.

AIC = −2 Log (Likelihood) + 2*T* (1)

where *T* is the number of parameters in the model. The likelihood is a calculation of the best-fitted model. Maximum values reveal a suitable fit.

Schwarz criterion (SIC) test is calculated to select the most adequate and suitable model among the determinate models. The adequate and appropriate model consists of the smallest of SIC and is approximately associated with the AIC.

SIC = −2 ln (Likelihood) + (*T* + *T* ln (*M*)) (2)

Here, *T* is the number of parameters in the model, and *M* describe the number of observations.

Hannan–Quinn criterion (HQC) is the opposite criterion for determining the best-fitted model to AIC and SIC.

HQC = −2 Log (Likelihood) + 2 (*T* + *T* ln (*M*)) (3)

*T* is the parameter number of the model. *M* describe the observation number.

Durbin–Watson Test (DW) is a test of statistical significance for calculating the linear relation between the adjacent residuals from a regression model. The hypothesis of the Durbin–Watson statistic is δ = 0 in the specification.

(4) $$Z_{t}=\delta \;Z_{t-1}+\varepsilon_{t}$$

Durbin–Watson (DW) is equal to 2, which expresses that correlation does not exist. DW < 2 shows positive correlation and ranging from 2 to 4 shows negative correlation, and the given series is correlated strongly if the value approaches approximately zero.

(5) $$\mathrm{The}\;\mathrm{Mean}\mathrm{Absolute}\mathrm{Error}\mathrm{MAE}=\frac{1}{m}\sum_{t=1}^{m}\left|\varepsilon_{t}\right|$$

With m observations, calculate the absolute deviation of predicted values from real ones, and known as Mean Absolute Deviation (MAD), is the magnitude of the total error of forecasting, and the effect of positive and negative errors does not cancel out.

(6) $$\mathrm{Mean}\mathrm{Absolute}\mathrm{Percentage}\mathrm{Error}\left(\mathrm{MAPE}\right)=\frac{1}{m}\sum_{t=1}^{m}\left|\frac{\varepsilon_{t}}{X_{t}}\right|\times 100$$

Describes the average absolute error percentage, which is scale measurement independent, and the direction of error is not specified. The extreme deviation is not penalized, and opposite-sign errors do not offset each other.

(7) $$\mathrm{Root}\mathrm{Mean}\mathrm{Squared}\mathrm{Error}\left(\mathrm{RMSE}\right)=\sqrt{\frac{1}{m}\sum_{t=1}^{m}\varepsilon_{t}^{2}}$$

Express the average squared deviation of predicted values and alternate signed error; do not offset one from another. The adequacy of the measurement errors is the smallest, and it receives a good result, with 0.1 MAPE achieved.

(8) $$Theil’s\;U\text{-}Statistics\;\left(U\right)=\frac{\sqrt{\frac{1}{m}\sum_{t=1}^{m}\xi_{t}^{2}}}{\sqrt{\frac{1}{m}\sum_{t=1}^{m}{g_{t}}^{2}}\sqrt{\frac{1}{m}\sum_{t=1}^{m}{Z_{t}}^{2}}}\;\;\;\;\;0\leq U\leq 1$$

Here *g_t_* denotes the forecasted value and *Z_t_* describes the value of actual observations. *U* is the normalized calculation of the total forecast error. If *U* is equal to 0 then, it reveals the perfect fit.

### 4.2. Test Normality

The normality test evaluates whether the data under consideration follows a normal distribution, primarily using skewness and excess kurtosis as indicators. Values near zero suggest normality. The Jarque–Bera test, which combines skewness and kurtosis measures, is employed to statistically assess deviations from normality.

(9) $$\mathrm{The}\;\mathrm{skewness}\;\mathrm{is}\;\mathrm{the}\;\mathrm{degree}\;\mathrm{of}\;\mathrm{asymmetry}\;\;\;\mathrm{Skewness}=\frac{\sum_{i=1}^{m}{\left(Z_{i}-\bar{Z}\right)}^{3}}{(m-1)T^{3}}$$

where $\bar{Z}$ represents the mean and *T* is the standard deviation and the number of values is represented by *m*. A skewness approaching zero shows that the dataset is symmetrically distributed. A skewness value greater than zero indicates a positive skew; otherwise, it indicates a negative skew.

The measurement of Kurtosis is the degree of peakness of the dataset, which is described as

(10) $$\mathrm{Kurtosis}=\frac{\sum_{i=1}^{m}{\left(Z_{i}-\bar{Z}\right)}^{4}}{(m-1)T^{4}}$$

where $\bar{Z}$ is the mean, *T* is the standard deviation, and *m* is the number of datasets. Kurtosis has a standard value of 3 with a normal distribution. If it is equal to 3, it is called mesokurtic, greater than 3 shows leptokurtic, and otherwise it is platykurtic.

Jurque–Bera Statistics (JBS) test is recognized with the dataset normality, with skewness equal to zero and surplus kurtosis also approximately equal to zero. The formation of the Jurque–Bera test is defined as

(11) $$\mathrm{Jurque}\text{-}\mathrm{Bera}\;\mathrm{test}=\frac{n{\left(Skewness\right)}^{2}}{6}+\frac{n{\left(Kurtosis-3\right)}^{2}}{24}$$

Jurque–Bera test statistics are nearly closed to Chi-squared distribution with two degrees of freedom. Null hypothesis (H_O_) is a normal distribution with zero skewness and surplus zero kurtosis (which is the same as a kurtosis is 3). Alternate hypothesis (H_A_) of given data is not normally distributed.

### 4.3. Autoregressive AR(p) Through GARCH Model

The autoregressive process (AR), originally introduced by Yule [42], models a variable as a weighted sum of its previous values plus a white noise term. The generalized AR(p) process of lag order *p* is defined as follows:

(12) $$Z_{t}=\alpha_{1}\;Z_{t-1}\;+\;\alpha_{2}\;Z_{t-2}\;+\;\ldots \;+\;\alpha_{p}Z_{t-p}\;+\;\tau_{t}$$

white noise ( τ *_t_*) with mean E ( τ *_t_*) = 0, variance Var ( τ *_t_*) = σ^2^ and Cov ( τ *_t_*_−*s*_, τ *_t_*) = 0, if *q* ≠ 0. For every *t* it is supposed that τ *_t_* is independent of the *Z_t_*_−1_, *Z_t_*_−2_, …… with *Z_q_* for each *q* < *t*.

The GARCH (1,1) model was first introduced by [43], while the generalized GARCH (*p*,*q*) stochastic volatility framework was later developed in [44,45]. As an extension of autoregressive conditional heteroskedasticity, the GARCH model effectively captures time-varying volatility and is well-suited for modeling volatility clustering and forecasting comparative variance [46]. The GARCH (1,1) specification is particularly noted for its superior predictive performance compared to traditional models [47] and is widely applied in multi-step forecasting across various domains, including financial and economic time series analysis. The GARCH model for the process *Z_t_* is defined as follows:

(13) $$Z_{t}=\;\sigma_{t}\epsilon_{t}$$

where $\sigma_{t}$ known as the conditional standard deviation and $\in_{t}\sim \;IID(0,\;1)$ . IID(0, 1) is defined as a sequence in statistical analysis that are independent and random variables identically distributed with mean 0 and variance 1.0.

The process is described as

(14) $$\sigma_{t}^{2}=\delta +\beta \;\tau_{t-1}^{2}+\gamma \;\sigma_{t-1}^{2}\;\mathrm{with}\;\delta +\beta +\gamma \;\geq 0$$

GARCH model is a covariance-stationary process that shows that its mean and variance do not change with respect to time. *δ* is constant and *β*, *γ* are GARCH coefficients and a white noise process if and only if *β* + *γ* < 1. The variance of the covariance-stationary process is calculated as follows:

(15) $$\mathrm{Var}\left(X_{t}\right)=\frac{\delta }{1-\beta -\gamma }$$

GARCH process is stationary with stationarity conditions. In the GARCH (1,1) process frequently follow leptokurtic which is kurtosis greater than 3 that represent the behavior of a heavy tailed. The AR(p)–GARCH (1,1) model is described as follows:

(16) $$Z_{t}=\alpha_{1}\;Z_{t-1}+\alpha_{2}\;Z_{t-2}+\ldots +\alpha_{p}\;Z_{t-p}+\tau_{t}$$

(17) $$t=\sigma_{t}\epsilon_{t}\;\mathrm{with}\;\in_{t}\sim \;IID(0,\;1)$$

(18) $$\sigma_{t}^{2}=\delta +\beta_{1}\tau_{t-1}^{2}+\beta_{2}\tau_{t-2}^{2}+\cdots +\beta_{p}\tau_{t-p}^{2}+\gamma \;\sigma_{t-1}^{2}$$

where E( τ *_t_*) = 0, variance Var ( τ *_t_* | $\tau_{t-1}^{2},\;\tau_{t-2}^{2}\ldots$ ) = *σ*^2^, and Cov( τ *_t_*_−*q*_, τ *_t_*) = 0, if *q* ≠ 0.

The Box–Jenkins method with GARCH process is used to select models, to analyze the models and to predict the seven attempts dataset.

## 5. Results and Discussion

**Figure 6:**
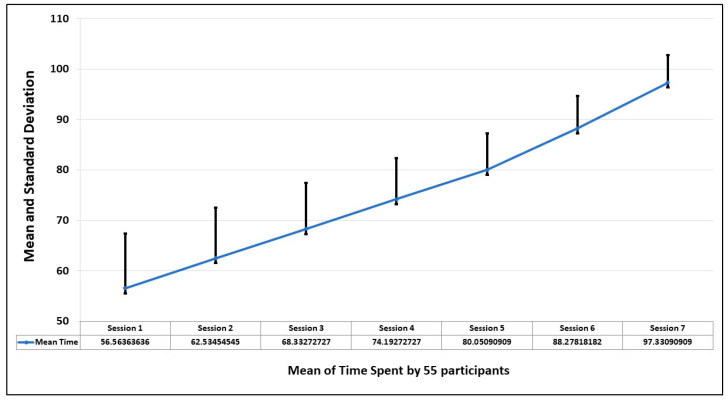
Mean and standard deviation of time of 55 participants in 7 sessions.

To study the fear levels of around 55 people, both men and women, aged from 18 to 35 years, took part in trials. All participants have completed the three stages of the application as shown in Figure 6. To analyze the fear level, three parameters are considered: time (time spent in completing a level and stage), head movement, and heart rate.

Participants’ FSQ scores ranged from *X* to *Y* (*M* = $\bar{X}$ , SD = $\bar{Y}$ ), with 60% scoring in the moderate-to-high fear range. This validated the relevance of the VRET intervention across a continuum of spider-fearful responses.

### 5.1. Time

**Figure 7:**
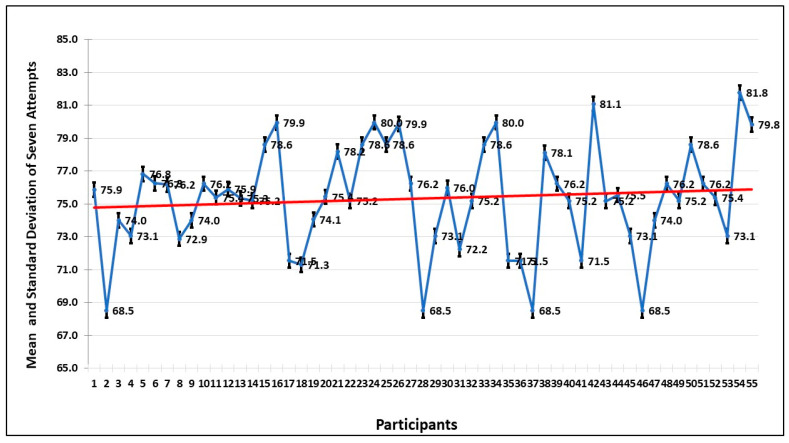
Trend of mean and standard deviations of time by the 55 participants in 7 sessions. The red line shows the mean value, and the blue line shows the range of values over a session.

The first parameter to observe the behavioral pattern of participants is the time spent to complete the session. This is analyzed by finding the standard deviation in seven sessions. The graph of Figure 7 shows that the average time improves in every session, which means as the person becomes familiar with the environment, the comfort level increases and the fear level decreases.

Figure 3 shows the mean and standard deviation of the time of seven sessions taken by each participant. The trend also shows that by spending more time, the fear level decreases.

The normality test and descriptive analysis of the mean dataset of time in all seven sessions are shown in Table 1. Except in session 4, which has negative skewness, all other sessions show positive skewness (right tail), which shows that events, savings, and forecasts are taken. Sessions 1 to 4 show platykurtic distribution, which represents a flat tail (peakness), whereas sessions 5 and 6 follow leptokurtic. The null hypothesis is rejected in the Jarque–Bera test, which indicates that attempts are normally distributed.

Table 2 summarizes the diagnostic results for AR(p)–GARCH (1,1) models across all seven sessions. Model adequacy was assessed using standardized residuals, the Lagrange Multiplier test for heteroskedasticity, and the Ljung–Box test for autocorrelation. R^2^ values indicated strong model fit, while Durbin–Watson statistics below 2 suggested positive autocorrelation. Model selection was guided by AIC, BIC, HQC, and log-likelihood values, with the AR(1)–GARCH (1,1) model identified as the best fit for most sessions.

Table 3 describes the prediction evolution and GARCH model equation = *α* + *β* Res^2^ + *γ*GARCH, where α is a constant and *β* and *γ* are GARCH coefficients. Table 3 also depicts the evolution of forecasting for each session. The MAE is the lowest value among RMSE and MAPE of all the attempts. Theil’s U-statistic is also used for the evolution of forecasting of time spent on each attempt, and it is equal to 0, which describes the best-fitted model and is correlated to the previous one.

### 5.2. Head Movement

**Figure 8:**
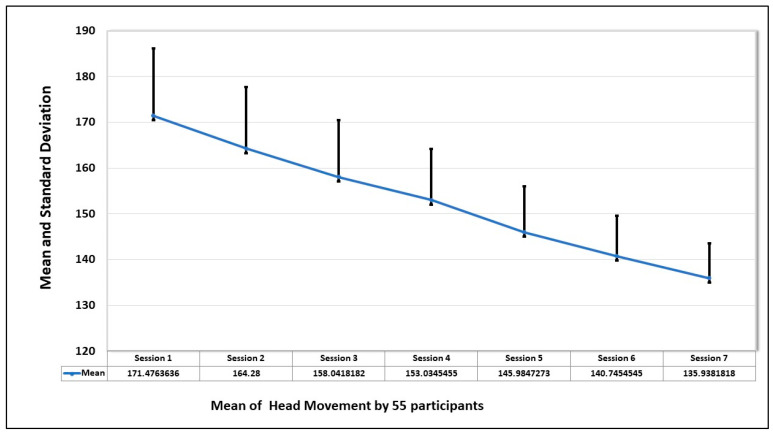
Mean and standard deviation of head movement of all the participants in seven sessions.

To further investigate the anxiety pattern of participants, the movement of the head during the seven sessions is examined. The graph of Figure 8 shows the average of the head movements, and it is evident that the head movement is high in session 1 and decreases in every session. This shows that when the participant gets familiar with the environment, the fear level decreases, and that shows they are more focused on completing the tasks.

**Figure 9:**
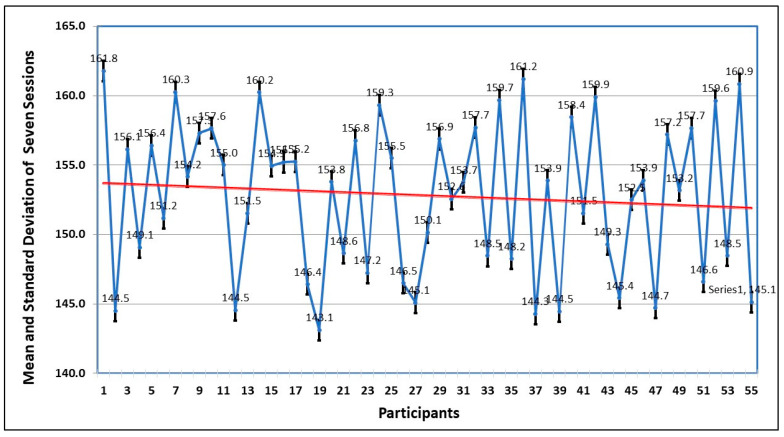
Trend of mean and standard deviations of head movement by the 55 participants in 7 sessions. The red line shows the mean value, and the blue line shows the range of values over a session.

The mean and the standard deviation in seven sessions by all the participants are shown in Figure 9. The trend also confirms what has been deduced from the graph, that the repeated exposure to the VRET environment decreases the anxiety levels, and they focus more on dealing with the fear stimulus, instead of avoiding it.

For the head movement in seven sessions by the participants, the normality test and descriptive analysis of the mean dataset are shown in Table 4. All sessions have negative skewness, which represents the risk of left tail events and unpredictability, and are platykurtic, which represents that flat tail (peakness). The null hypothesis is rejected in the Jarque–Bera test, which indicates that attempts are normally distributed.

Table 5 characterizes the most appropriate model for each level of seven attempts, with the smallest value of estimation being used to select the AR(p)–GARCH (1,1) model. The criteria of selection are the same as for time spent. As the AIC, SIC, HQC, and Durbin–Watson (DW) test denote the maximum attempt, the best-fitted model is an AR(1)–GARCH (1,1) model. R2 describes that the data set values in each attempt are consistent with each other.

Table 6 also depicts the evolution of forecasting for each attempt analysis. MAE is the lowest value among RMSE and MAPE of all the attempts. Theil’s U-statistic is also used for the evolution of forecasting of time spent on each attempt and is equal to 0, which describes the best-fitted model and is correlated to the previous one.

### 5.3. Heart Rate

**Figure 10:**
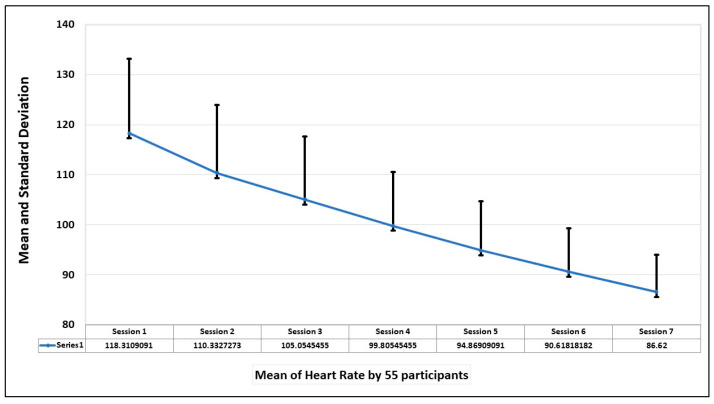
Mean and standard deviation of heart rate of all the participants in seven sessions.

The final evaluation parameter is the heart rate of the participants during the sessions. The mean and standard deviation of the heart rate of 55 participants in seven sessions is shown in the graph in Figure 10. The graph shows that heart rate was highest when the first session was taken but decreased with the increase in exposure to the fear stimulus.

**Figure 11:**
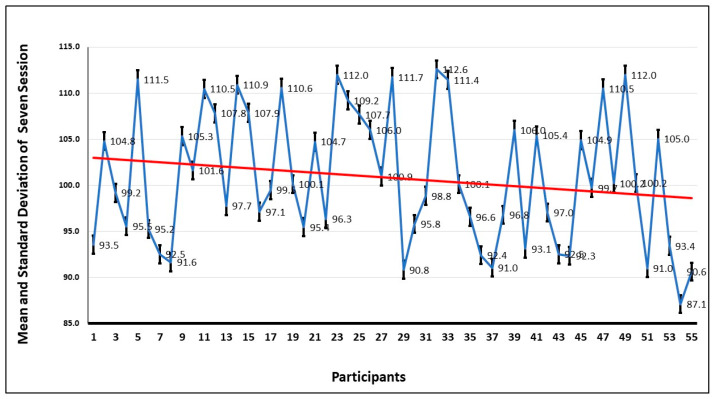
Trend of mean and standard deviations of heart rate by the 55 participants in 7 sessions. The red line shows the mean value, and the blue line shows the range of values over a session.

Also, from the graph of Figure 11, which depicts the average heart rate of each participant in seven sessions, the trend is that the heart rate improves with the increase in exposure to the spiders.

Table 7 describes the normality test and descriptive analysis for the mean dataset of heart rate in seven sessions. The majority of sessions have positive skewness (right tail), which shows that events, savings, and forecasts are present, except for the first and fifth sessions, which have negative skewness and are platykurtic, representing a flat tail (peakness). The null hypothesis is rejected in the Jarque–Bera test, which indicates that attempts are normally distributed.

Table 8 describes the best fitted model for each session of the mean heart rate with the lowest value of AIC, SIC, and HQC and Durbin–Watson (DW) test with the same criteria.

Table 9 also depicts the evolution of forecasting for each session analysis. MAE is the lowest value among RMSE and MAPE of all the attempts. Theil’s U-statistic is used for the evolution of forecasting of the time spent on each attempt. Theil’s U-statistic is equal to 0, which describes the best-fitted model and is correlated to the previous one.

Prior to model fitting, stationarity was confirmed using the Augmented Dickey–Fuller (ADF) test. The presence of ARCH effects, confirmed via Lagrange Multiplier (LM) tests, supported the use of GARCH to capture time-dependent variance in response to fear-inducing stimuli.

A subset of participants (*N* = 55) completed the Fear of Spiders Questionnaire (FSQ) pre- and post-VRET intervention. Table 10 presents the change in FSQ scores by baseline fear severity. All subgroups demonstrated statistically significant reductions in fear levels. Participants with high baseline fear (FSQ ≥ 80) showed the greatest absolute improvement (−33.5 points, *p* < 0.001), followed by those with moderate (−21.6) and low-moderate fear (−9.3). The overall sample exhibited a 34.2% reduction in spider-related fear, supporting the efficacy of the VRET protocol in addressing arachnophobia symptoms.

### 5.4. Inferential Statistics: Hypothesis Testing for Key Indicators

To supplement the AR-GARCH analysis, paired-sample *t*-tests were conducted to assess the impact of the VET system on key behavioral and physiological indicators across sessions. Results revealed statistically significant reductions across all measures. FSQ scores decreased markedly from pre- to post-intervention, indicating a substantial reduction in self-reported fear levels (t(54) = 41.41, *p* < 0.001, d = 5.58). Heart rate also dropped significantly (t(54) = 35.29, *p* < 0.001, d = 4.76), reflecting reduced physiological arousal. Time spent in exposure scenarios declined (t(54) = 38.28, *p* < 0.001, d = 5.16), and head movement variability showed a notable decrease (t(54) = 20.43, *p* < 0.001, d = 2.76), indicating improved emotional stability and engagement as discussed in Table 11. These results confirm the effectiveness of the VET intervention in alleviating arachnophobia symptoms across multiple dimensions.

In addition to statistical significance, effect sizes were calculated using Cohen’s d to quantify the magnitude of the intervention effects. Results showed a very large effect in the reduction in FSQ scores from pre- to post-intervention (d = 5.58), indicating a strong improvement in self-reported spider fear symptoms. Heart rate reductions from session 1 to session 7 also demonstrated a large effect size (d = 4.76), while reductions in time spent in fear-inducing zones (d = 5.16) and head movement variability (d = 2.76) further supported the system’s impact. These effect sizes confirm that the observed changes are not only statistically significant but also practically meaningful in a behavioral intervention context. The large effect sizes observed across behavioral and physiological measures (Cohen’s d > 2.5) emphasize the practical utility of the VET system for reducing spider fear symptoms, beyond mere statistical significance.

### 5.5. Why This Application—Complementing Prior RCTs

Rigorous trials already show that automated VR therapy can reduce specific-phobia symptoms (e.g., automated acrophobia treatment with a virtual coach; automated VRET non-inferior to in vivo one-session treatment for spider phobia; self-guided smartphone/consumer VR programs). Those studies primarily establish efficacy and, in some cases, effectiveness at the level of session means and clinical endpoints. In contrast, our application targets (i) process-level dynamics—how reactivity settles during exposure—by quantifying within-session volatility (persistence, half-life) across heart rate, head movement, and time-on-task, and (ii) a reportable, physiology-aware adaptation rule (threshold micro-relaxations and paced step-ups) designed for standalone hardware. This combination addresses field-level calls to move beyond outcome means, to standardize physiology-informed titration, and to publish transparent control logic that others can deploy and audit. Our claims are therefore complementary to RCTs: we contribute mechanism-linked measurement and implementation detail needed for scale-up, not a replacement for trial-level efficacy evidence.

The design intentionally prioritizes symptom severity sampling to interrogate exposure process mechanisms and adaptive control under pragmatic conditions, a strategy that is increasingly endorsed by dimensional frameworks (e.g., HiTOP) when the goal is to understand change processes before scaling to clinic-diagnosed RCTs.

Our claims are deliberately modest: conditional variance modeling offers an additional lens on exposure processes—how reactivity settles within sessions—rather than a replacement for mean-focused analyses. Evidence of preconditioned fit gains, elimination of residual volatility, and incremental predictive value (replicated with simple proxies) indicates added, decision-relevant information without reliance on a single model family.

Conditional variance metrics are most informative when exposures produce detectable volatility clustering and when early process markers could guide adaptive pacing (e.g., step-up/hold rules). Where volatility is absent or the analytic goal is exclusively mean change, standard RM-ANOVA/LMM or LMM with variance functions is sufficient.

### 5.6. Limitations

A key limitation is the absence of structured clinical diagnosis; consequently, findings generalize to high-spider-fear adults rather than to patients meeting *DSM-5-TR* criteria for specific phobia, which require clinically significant distress/impairment and persistent, situation-specific fear/avoidance verified by a clinician. This sampling strategy matches many prior VRET process and feasibility studies and reflects our mechanistic focus on within-session dynamics and physiology-aware adaptation. Future trials should replicate these effects in clinician-diagnosed samples using standardized interviews and functional impairment measures.

### 5.7. Present Value Independent of Future Work

While future clinician-diagnosed RCTs are important, the current manuscript stands on its own by (i) introducing interpretable process metrics that characterize how exposure settles within sessions; (ii) demonstrating incremental predictive utility beyond means; (iii) providing robustness evidence via a specification curve and proxy replications; and (iv) publishing transparent, device-agnostic control logic that others can implement immediately. These contributions improve measurement, prediction, and implementation today; future trials will test clinical generalizability, not supply the core value of this work.

## 6. Conclusions

This study reports a pragmatic evaluation of a standalone virtual reality exposure workflow for adults with high spider fear across seven sessions. As expected, and consistent with the literature, fear indices decreased over time with repeated exposure. Our contribution is incremental and implementation-oriented rather than a claim of superior efficacy: we (i) make within-session processes measurable by operationalizing settling dynamics of arousal/avoidance and (ii) specify a transparent, device-agnostic micro-relaxation rule that can be audited and replicated in low-touch deployments. We explicitly acknowledge methodological boundaries that constrain causal and clinical claims. Participants were not clinician-diagnosed, there was no randomized control or comparator, and outcomes were not anchored with a behavioral approach test; hence, results generalize to high-fear, non-diagnosed adults and should not be interpreted as trial-level evidence of treatment superiority. The design was intended to probe process measurement and implementation feasibility, not to substitute for clinician-diagnosed RCTs.

The time series analysis is positioned as a complement, not a replacement, for standard psychological models. Conditional variance (AR(p)–GARCH) descriptors were used exploratorily to summarize within-session volatility (e.g., persistence, half-life)—quantities that mean-based summaries do not provide. These descriptors are offered as optional process metrics to aid interpretation of how reactivity settles during exposure; the practical guidance (signals to log, trigger thresholds, pacing logic) does not depend on the choice of GARCH and can be implemented using simple rolling-window summaries where preferred. To address concerns about presentation clarity, the revised reporting centers on decision-relevant estimates (point estimates with 95% CIs) with four focused figures (flow/protocol; outcome trajectory; process trajectories; process–outcome association) and three concise tables (sample characteristics; primary outcomes; process metrics).

Practically, the manuscript contributes a reproducible control recipe—signal baselining, thresholding, brief down-regulation prompts, and conservative step-up/hold rules—that can be adopted in therapist-guided or automated VRET on commodity/standalone hardware. These elements aim to standardize physiology-aware titration and support early identification of participants who may require slower pacing or additional support. In sum, this work should be read as a measured addition to the VRET evidence base: it does not claim novelty in demonstrating symptom reduction, nor superiority over established methods. Instead, it offers a clearer way to measure moment-to-moment exposure dynamics and a transparent, implementable adaptation rule that services and developers can use now. Definitive questions of clinical efficacy and generalizability belong to future clinician-diagnosed, controlled trials that embed these process metrics and control logic within preregistered protocols.

## Figures and Tables

**Table 1:** Test of normality of arachnophobia patients for the mean time in 7 sessions.

Attempts	Mean	Median	S. Deviation	Skewness	Kurtosis	Jarque–Bera Test
1	56.56	56.15	3.68	0.75	2.99	5.38
2	62.53	62.20	2.83	0.34	2.08	3.06
3	68.33	68.20	3.44	0.32	2.546	1.48
4	74.19	74.55	3.77	−0.31	2.00	3.25
5	80.05	79.70	4.26	0.11	1.82	3.35
6	88.27	88.75	6.58	0.77	4.74	12.67
7	97.331	93.85	10.32	1.19	3.24	13.52

**Table 2:** Test diagnostic of AR(p)–GARCH (1,1) process of arachnophobic patients for the mean time in sessions (1st to 7th).

Session	AR(p)–GARCH (1,1)	R-Square	Adjusted R-Square	SE of Regression	Log Likelihood	AIC	SIC	HQC	Durbin–Watson
1	AR (1)	0.12	0.10	3.48	−143.43	5.30	5.48	5.37	1.64
2	AR (1)	0.27	0.25	2.44	−125.33	4.65	4.83	4.72	1.86
3	AR (1)	0.15	0.14	3.18	−143.20	5.29	5.47	5.36	1.91
4	AR (6)	−0.03	−0.05	3.88	−146.70	5.41	5.59	5.48	1.52
5	AR (5)	0.01	−0.00	4.28	−157.17	5.79	5.97	5.86	1.48
6	AR (13)	0.04	0.02	6.49	−178.74	6.56	6.74	6.63	1.52
7	AR (3)	−0.05	−0.07	10.70	−200.68	7.34	7.52	7.41	1.58

**Table 3:** Forecast evolution of AR(p)–GARCH (1,1) process of arachnophobia patients for the mean time in sessions (1st to 7th).

Session	AR–GARCH	RMSE	MAE	MAPE	U Test	$\mathrm{GARCH}=\alpha +\beta {\mathrm{Res}}^{2}+\gamma$ GARCH
1	AR (1)	3.71	2.68	4.64	0.03	1.06 − 0.21*R* + 1.12*G*
2	AR (1)	2.84	2.34	3.76	0.02	0.41 − 0.17*R* + 1.10*G*
3	AR (1)	3.44	2.56	3.75	0.02	8.24 − 0.09*R* + 0.25*G*
4	AR (6)	3.72	3.02	4.13	0.02	7.71 − 0.22*R* + 0.60*G*
5	AR (5)	4.23	3.44	4.291	0.02	15.51 − 0.45*R* + 0.59*G*
6	AR (13)	6.47	4.40	4.92	0.03	19.36 − 0.11*R* + 0.61*G*
7	AR (3)	10.08	6.42	6.11	0.05	11.26 − 0.12*R* + 0.98*G*

**Table 4:** Test of normality of arachnophobia patients for the mean head movement in 7 sessions.

Attempts	Mean	Median	S. Deviation	Skewness	Kurtosis	Jarque–Bera Test
1	171.47	172.50	5.67	−0.52	2.68	2.79
2	164.28	164.20	6.36	−0.57	2.31	4.11
3	158.04	157.20	6.03	−0.17	2.08	2.19
4	153.03	152.30	5.91	−0.13	2.21	1.59
5	145.98	147.20	6.28	−0.04	2.02	2.17
6	140.74	141.20	6.91	−0.39	2.82	1.49
7	135.93	138.60	6.14	−0.70	2.72	4.79

**Table 5:** Test diagnostic of AR(p)–GARCH (1,1) process of arachnophobia patients for the mean of head movement in 7 sessions.

Attempts	AR(p)–GARCH (1,1)	R-Square	Adjusted R-Square	SE of Regression	Log Likelihood	AIC	SIC	HQC	Durbin–Watson
1	AR (4)	0.10	0.08	5.43	−164.44	6.16	6.34	6.23	1.98
2	AR (1)	0.02	0.00	6.34	−172.89	6.46	6.65	6.53	1.77
3	AR (1)	0.07	0.06	5.85	−172.37	6.45	6.63	6.52	1.93
4	AR (1)	0.06	0.04	5.79	−168.29	6.30	6.48	6.37	1.69
5	AR (16)	0.09	0.07	6.04	−172.57	6.45	6.63	6.52	1.78
6	AR (1)	0.12	0.11	6.51	−175.77	6.57	6.75	6.64	1.64
7	AR (4)	0.03	0.01	6.09	−169.79	6.35	6.53853	6.42	1.54

**Table 6:** Forecast evolution of AR(p)–GARCH (1,1) process of arachnophobia patients for the mean of head movement in seven sessions.

Attempts	AR–GARCH	RMSE	MAE	MAPE	U Test	$\mathrm{GARCH}=\alpha +\beta {\mathrm{Res}}^{2}+\gamma$ GARCH
1	AR (4)	5.72	4.59	2.71	0.01	1.70 − 0.16*R* + 1.13*G*
2	AR (1)	6.32	5.22	3.23	0.01	5.60 − 0.25*R* + 1.11*G*
3	AR (1)	5.82	4.75	3.01	0.01	21.29 − 0.30*R* + 0.64*G*
4	AR (1)	5.51	4.63	3.05	0.01	4.10 − 0.29*R* + 1.13*G*
5	AR (16)	7.06	5.82	4.05	0.02	7.59 − 0.32*R* + 1.07*G*
6	AR (1)	6.64	5.22	3.76	0.02	7.38 − 0.30*R* + 1.10*G*
7	AR (4)	6.25	4.94	3.75	0.02	17.82 − 0.16*R* + 0.64*G*

**Table 7:** Test of normality of arachnophobia patients for the mean heart rate in 7 sessions.

Attempts	Mean	Median	S. Deviation	Skewness	Kurtosis	Jarque–Bera Test
1	118.31	119.70	6.83	−0.15	2.33	1.23
2	110.33	108.60	6.94	0.15	1.70	4.08
3	105.05	103.20	6.95	0.11	1.71	3.92
4	99.80	100.10	7.54	0.17	1.86	3.22
5	94.86	96.10	9.26	−0.09	2.08	1.99
6	90.6	90.50	9.400	0.05	1.85	3.06
7	86.62	85.40	9.26	0.10	1.86	3.08

**Table 8:** Test diagnostic of AR(p)–GARCH (1,1) process of arachnophobia patients for the mean heart rate in 7 sessions.

Attempts	AR(p)–GARCH (1,1)	R-Square	Adjusted R-Square	SE of Regression	Log Likelihood	AIC	SIC	HQC	Durbin–Watson
1	AR (2)	−0.00	−0.02	6.92	−178.11	6.65	6.84	6.72	1.75
2	AR (3)	−0.11	−0.14	7.42	−181.71	6.78	6.97	6.86	1.61
3	AR (1)	−0.14	−0.166	7.51	−182.29	6.81	6.99	6.88	1.38
4	AR (4)	0.01	−0.00	7.57	−185.17	6.91	7.09	6.98	1.82
5	AR (7)	−0.04	−0.06	9.53	−194.88	7.26	7.45	7.33	1.62
6	AR (12)	−0.00	−0.02	9.50	−197.04	7.34	7.52	7.41	1.49
7	AR (6)	0.00	−0.01	9.34	−195.90	7.03	7.48	7.37	1.40

**Table 9:** Forecast evolution of AR(p)–GARCH (1,1) process of arachnophobic patients for the mean heart rate, seven sessions.

Attempts	AR–GARCH	RMSE	MAE	MAPE	U Test	$\mathrm{GARCH}=\alpha +\beta {\mathrm{Res}}^{2}+\gamma$ GARCH
1	AR (2)	6.94	5.86	5.02	0.02	22.17 − 0.39*R* + 0.90*G*
2	AR (3)	7.78	6.25	5.50	0.03	27.94 − 0.32*R* + 0.79*G*
3	AR (1)	7.03	5.86	5.50	0.04	18.95 − 0.31*R* + 0.962*G*
4	AR (4)	7.61	6.37	6.35	0.03	10.64 − 0.33*R* + 1.12*G*
5	AR (7)	9.42	7.83	8.30	0.04	120.16 + 0.34*R* − 0.71*G*
6	AR (12)	9.70	8.09	9.06	0.05	17.00 − 0.30*R* + 1.09*G*
7	AR (6)	9.27	7.82	9.17	0.05	18.39 − 0.35*R* + 1.13*G*

**Table 10:** Change in FSQ scores by baseline fear severity.

Fear Level Group	*N*	Pre-VRET FSQ Mean (SD)	Post-VRET FSQ Mean (SD)	Mean Difference	% Reduction	t/z Value	*p*-Value
High (FSQ ≥ 80)	18	88.2 (±4.5)	54.7 (±6.2)	−33.5	38.0%	t = 14.23	<0.001
Moderate (FSQ 50–79)	24	67.8 (±6.9)	46.2 (±7.8)	−21.6	31.8%	t = 11.89	<0.001
Low-Moderate (FSQ 40–49)	13	44.2 (±3.2)	34.9 (±4.1)	−9.3	21.0%	t = 6.02	<0.001
Total Sample	55	72.3 (±10.8)	47.6 (±11.5)	−24.7	34.2%	t = 13.41	<0.001

**Table 11:** Paired *t*-test results table.

Measure	Pre-Mean (SD)	Post Mean (SD)	t-Value	*p*-Value	Cohen’s d
FSQ Score	70.26 (9.82)	45.61 (9.97)	41.40	1.34 × 10^−42^	5.58
Heart Rate	104.33 (6.31)	88.00 (7.08)	35.29	5.56 × 10^−39^	4.75
Time Spent	177.72 (28.67)	114.59 (29.85)	38.28	8.13 × 10^−41^	5.16
Head Movement Variability	14.38 (2.71)	10.74 (3.03)	20.43	4.45 × 10^−27^	2.75

## Data Availability

Data will be made available on request.
